# Is a hybrid of online and face-to-face services feasible for audiological rehabilitation post COVID-19? Findings from three public health patients

**DOI:** 10.4102/sajcd.v69i2.907

**Published:** 2022-08-17

**Authors:** Nuha Khatib, Vera-Genevey Hlayisi

**Affiliations:** 1 Department of Health and Rehabilitation Sciences, Faculty of Health Sciences, University of Cape Town, Cape Town, South Africa

**Keywords:** tele-audiology, hybrid, COVID-19, public health, auditory training, hearing aids, adult, South Africa

## Abstract

**Background:**

The global coronavirus disease 2019 (COVID-19) pandemic has pushed many audiologists to incorporate remote service delivery methods to adhere to mandatory health and safety protocols. The use of tele-audiology for audiological rehabilitation may provide a sustainable, cost-effective modality to suit the existing need, particularly in low-resourced countries.

**Objectives:**

This study aimed to investigate the feasibility of implementing a hybrid tele-rehabilitation programme in a South African public health context. An online auditory training (AT) programme was used to determine (1) compliance, (2) clinical benefit, (3) participant experience and (4) costs.

**Method:**

A convergent mixed methods design with a feasibility approach was utilised. Data collection was done through questionnaires, in-booth assessments, online AT, and face-to-face interviewing. Participants undertook online AT over 4 weeks. For pre- and post-online AT, the Abbreviated Profile of Hearing Aid Benefit (APHAB), QuickSIN, entrance and exit questionnaires, interviews and a system usability scale were administered.

**Results:**

Key findings of this study included (1) a high compliance rate (84.82%) with minimal clinician contact time at 3 h 25 min over 5–6-weeks; (2) improvement in perceived hearing aid (HA) benefit, and improvement in listening skills; (3) reported positive experiences; and (4) minimal programme costs at an average of R1350.00 per participant.

**Conclusion:**

The results showed positive indicators that the use of hybrid tele-rehabilitative strategies may provide a viable alternative to the traditional face-to-face modality. The hybrid approach showed clinical benefits, cost-effectiveness, minimal contact time as well as COVID-19 compliance. Further large-scale research is still needed.

## Introduction

One of the negative effects of the coronavirus disease 2019 (COVID-19) lockdown protocol in South Africa was the temporary halting of nonessential services, with essential services referring mainly to hospital care, police forces and grocery store operation (Banerjee, Robinson, Sathian, & Van Teijlingen, 2020). Many audiologists sought alternative service delivery modalities to protect their patients and themselves against COVID-19, while providing a high standard of care that ensured ethical testing, rehabilitation and treatment of audiology patients. The adoption of hybrid (online and face-to-face) tele-audiology was a way for audiologists to do this.

Many audiologists globally already use hybrid methods to provide hearing healthcare to a larger population (Ballachanda, 2019; Bush, Thompson, Irungu, & Ayugi, 2016; Havenga, Swanepoel, Le Roux, & Schmid, 2017; Ratanjee-Vanmali, Swanepoel, & Laplante-Lévesque, 2020). The successful implementation of audiological services using online methods lowers the risk of COVID-19 infection. This is particularly pertinent given the fact that a large proportion of the general audiology patient population is of geriatric age with probable comorbidities (e.g. high blood pressure and chronic respiratory disease) (Ko et al., 2020). The use of a hybrid model may also provide long-term benefits to practices (in relation to costs, clinician time and patient benefits) (Reis, Boisvert, Beedell, & Mumford, 2019; Tye-Murray, Spehar, Barcroft, & Sommers, 2017). Some of the challenges of online services, such as technology limitations (e.g. Internet-capable device access and technological incompatibility of older hearing aids [HAs]), patient readiness and the reliability of some online assessments (Ratanjee-Vanmali, Swanepoel, & Laplante-Lévesque, 2019; Ravi, Gunjawate, Yerraguntla, & Driscoll, 2018; Rutherford & Petersen, 2019), all of which may restrict many from taking full advantage of the opportunities.

### Feasibility of a hybrid tele-audiology model

A recent review by Tao et al. (2018) included international tele-audiology studies focusing on adult HA users and found that remote HA fittings and subsequent follow-ups were indeed feasible. However, they highlighted the need for further tele-audiology research, particularly including the delivery of rehabilitative strategies such as auditory training (AT). The current feasibility study aimed to contribute to the knowledge base on tele-rehabilitation applications involving AT provision for adult HA users.

Abrams, Bock and Irey (2015), evaluating Internet-based AT on new HA users, showed that the online platform presents a promising model for AT delivery for audiological rehabilitation but that using Internet-based AT to provide audiological rehabilitation still implied considerable administrative time and clinician contact with the patient. This is important to consider for the South African public health context, as clinician time is one of the limitations or barriers currently preventing the provision of comprehensive audiological rehabilitation beyond HA fitting such as AT (Chisolm et al., 2013; Dubno, 2013; Ferguson & Henshaw, 2015; Sweetow & Palmer, 2005).

The cost and workload aspects are important factors to consider with tele-audiology applications, together with the patient experience and satisfaction with the services provided, which should also be considered. A study conducted in South Africa concluded that the hybrid model has great potential and importance in influencing patient satisfaction and compliance (Ratanjee-Vanmali et al., 2020). Furthermore, some of their key findings indicated that patient satisfaction with hybrid services was very high (97%), and patients preferred some online services compared to traditional face-to-face services (Ratanjee-Vanmali et al., 2020). They also found that patients were highly likely to recommend hybrid services to friends and family (Ratanjee-Vanmali et al., 2020).

Strategies such as perceptual training (e.g. AT) after HA fitting are considered a critical aspect in comprehensive audiological rehabilitation. Many studies have shown that AT improves auditory skills in people with hearing loss (Humes et al., 2019; Santos, Marangoni, Andrade, Prestes, & Gil, 2014; Tye-Murray et al., 2017). The training of cognitive skills which underlie successful speech perception may provide more generalisable outcomes and benefits for HA users, such as more effective communication (Ferguson & Henshaw, 2015). Furthermore, a recent systematic review showed that some AT programmes may have positive effects on overall cognition in older adults with hearing loss (Lawrence et al., 2018).

South African hearing healthcare provision for public health patients with hearing loss is marred with challenges, which include a lack of qualified audiologists, a lack of contextually relevant rehabilitative tools and limited provision of specialised rehabilitation such as AT. The lack of resources combined with the large patient load has resulted in a delay of service provision and hindered the successful implementation of comprehensive audiological rehabilitation in the public health sector (Pascoe, Rogers, & Norman, 2013; Pienaar, Stearn, & Swanepoel, 2010; Rutherford & Petersen, 2019). There is a need for the investigation of tele-rehabilitation in South African public healthcare to assist in the limited comprehensive audiological rehabilitation provision (Ratanjee-Vanmali et al., 2019; Scott & Mars, 2015; Swanepoel, 2016).

### Study rationale

With the growing impact of tele-audiology on healthcare in low-resource contexts, there is a great need for investigation into feasibility of tele-rehabilitation to assist in the limited hearing healthcare provision (which currently does not typically include AT) and provide COVID-19 safe services. This study was, to the knowledge of the researcher, the first to address the research question: ‘Is the implementation of a tele-rehabilitation programme for adult HA users feasible in a South African public health context?’.

## Research methodology

### Aim

To investigate the feasibility of implementing an online AT programme within a South African public health context.

### Objectives

The objectives of the study were to:

1. determine participants’ compliance to an online AT programme over 4 weeks with respect to the:
  - number of sessions spent on the programme per week
  - frequency of interactions and contact time between clinician and each participant
  - number of participants who complete the prescribed sessions versus any withdrawals
2. determine the effect of the online AT programme on participants’ speech perception in noise (pre- and post-administration of the online AT programme)
3. assess participants’ experience and benefit of online AT through interviewing and self-report questionnaires
4. estimate the costs around online AT programme implementation (online access, participant data usage, travel costs).

### Research design

A feasibility study using a convergent mixed methods design (quantitative and qualitative) was utilised. Feasibility studies allow the investigation and inclusion of data from different aspects of an intervention modality (e.g. patient performance, patient experience, clinical benefit and intervention expenses) (Orsmond & Cohn, 2015). The quantitative methods included assessments of auditory skills (speech perception and online AT tasks) and Likert questionnaires and were used within a single group pretest–post-test design. The qualitative methods included semistructured interviews and were used within a generic qualitative research design.

### Study setting

The current study was conducted in Cape Town at Groote Schuur Hospital (GSH). The hospital has Ear, Nose and Throat (ENT) and Audiology Departments. The audiology clinic provides services to both paediatric and adult patients. Services include diagnostic audiology testing, rehabilitative services and HA fittings. In alignment with the public health protocol, adult audiology patients at GSH usually receive one HA even with a bilateral hearing loss.

### Participants

#### Sampling

A nonprobability purposive sampling method was used. This sampling strategy was used to specifically select the participants, firstly, with a sensorineural hearing loss – because this specific patient was targeted by AT intervention (Brouns, El Refaie, & Pryce, 2011; Bryck & Fisher, 2012). Secondly, the method of AT delivery in the current study required participants to have access to an Internet-capable device. Finally, the language restriction on the selected AT programme was English. The full participant criteria are presented next.

##### Inclusion criteria

Hearing aid users:

- aged 19–59 years.
- with a mild to severe sensorineural hearing loss (unilateral or bilateral)
- with access to a smartphone, laptop, computer or tablet
- who could communicate in English
- who had at least 1 month experience of HA use (monaural or binaural).

##### Exclusion criteria

The following individuals were excluded from the study if they:

- were already receiving AT
- had a known cognitive impairment
- had a known literacy impairment
- had a bilateral flat profound sensorineural hearing loss.

#### Recruitment

The recruitment methods included both direct (telephonic calls) and indirect (recruitment posters) methods. Direct recruitment involved the researcher reviewing the audiology department’s HA patient folders to identify those who met the superficial study criteria – for example, age, hearing loss and language criteria. This was followed by telephonic contact to invite eligible participants. The eligible participants were firstly given an overview of the study and then the opportunity to ask questions, and if they were interested, matters regarding participation and consent were explained to them. Confidentiality was kept throughout the recruitment and data collection process as per research ethical guidelines (which included no sharing of medical results, training performance or any personal details of participants to parties outside of the research study).

**FIGURE 1:**
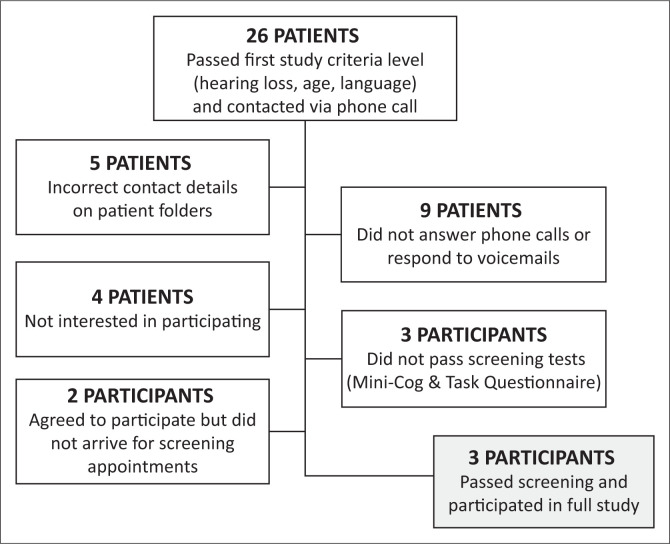
Participant recruitment.

A total of 26 patients were identified and passed the initial eligibility criteria (e.g. age, hearing loss, HA fitting, language and device access) for recruitment to this study. An in-person screening was conducted on those who agreed to participate in order to determine their full eligibility. The screening included a cognitive screener and a brief technological task ability questionnaire (details are given under the ‘Study materials’ section). After the screening and eligibility determination, there were initially five eligible participants. However, because of two participants not arriving for appointments, three participants were included in this feasibility study (see Figure 1). All included participants were women between the ages of 35 and 55 years, diagnosed with a bilateral hearing loss and fitted monaurally. The participants’ aided period (duration since fitting) ranged from 6 to 24 months.

### Study materials

- *Mini-Cog (screening tool 1):* paper-based standardised test used to distinguish between persons with dementia and persons without dementia (Borson, Scanlan, Brush, Vitaliano, & Dokmak, 2000). Used in the current study to screen for undetected cognitive impairment.
- *Task Ability Questionnaire (screening tool 2):* designed by the researchers to assess participants’ ability to perform the basic technological functions to use the online AT programme (intervention) (e.g. navigate to a website independently).
- *Listening and Communication Enhancement (intervention):* Listening and Communication Enhancement (LACE) online training platform (DeFauw & Henderson-Sabes, 2014). The LACE Online includes four different types of activities (e.g. word memory, rapid speech, speech in noise and competing speaker). The LACE is available in American and British English, which limits its applicability to South Africa. However, future South African AT tele-rehabilitation programmes may use the LACE Online platform design to build a more contextually relevant programme as it is the most user-friendly. The LACE Online includes a clinician monitoring function and a shorter programme moulded from years of research and HA user feedback to provide similar benefit as the original LACE, however, at a more accessible level (DeFauw & Henderson-Sabes, 2014). The LACE Online consists of 11 sessions, each approximately 25–45 min in length. Each session contains a combination of listening exercises. As patients progress through the 11 sessions, the difficulty of the level changes depending on their scoring. For example, if patients improve in their scores, the tasks become more challenging (lower signal-to-noise ratio [SNR]). The inverse is that if patients struggle, then tasks become easier (higher SNR). The LACE Online also administers the QuickSIN in the middle and at the end of the programme.
- *Quick Speech In Noise (QuickSIN):* a standardised test used to determine patients’ ability of understanding speech in noise. Patients are tested in an audiometric booth and presented with sentences in one speaker and white noise in the other. They are expected to repeat each sentence in the presence of the noise. According to the QuickSIN development team, Etymōtic Research (2001), the score obtained in the QuickSIN test is accurate to 2.7 decibels (dB) at a 95% confidence level. Although a QuickSIN assessment is included in the LACE Online programme, it was also conducted by the researcher in the current study in an audiometric booth pre- and post-intervention. In order to minimise the influence of the learning effect, the in-booth QuickSIN was administered with different wordlists for the pre- and post- assessments.
- *Abbreviated Profile of Hearing Aid Benefit (APHAB):* Likert-scale questionnaire which includes questions based on the HA user’s experience in specific listening situations (Cox & Alexander, 1995). The APHAB is separated into four subscales: Ease of Communication (EC), Reverberation (RV), Background Noise (BN) and Aversiveness (AV). Each statement or item in the APHAB is scored in a percentage. In all subscales an increase in percentage score indicates an improvement in perceived benefit. The APHAB was used as a formal means to measure perceived HA benefit pre- and post-intervention.
- *Entrance questionnaire:* Likert-scale questionnaire designed by the researchers to determine previous exposure to AT; anticipated motivation to complete the AT; preferred Internet-capable device; and preferred method of contact.
- *Exit questionnaire:* Likert-scale questionnaire designed by the researchers and questions were framed around programme accessibility and interface.
- *System Usability Scale (SUS):* The SUS (Brooke, 1996), is a 10-item Likert-scale questionnaire used to assess participants’ perceived usability of a programme (i.e. LACE Online). According to Brooke (1996), measuring usability should include (1) the effectiveness of the programme or system – the ability of participants to complete the programme; (2) efficiency – the resources needed and used to complete the programme; and (3) satisfaction – participants’ opinion and experience. The SUS was included because it is a simple standardised scale that has been widely used in research and is considered an intrinsic tool when assessing perceived usability (Lewis, 2018).
- *Interviews:* The inclusion of semistructured interviews (before and after AT) in the current feasibility study served to allow comprehensive inclusion of participant experiences and opinions, as well as to enable the researcher to build rapport with participants. Entrance interviews determined participants’ case history, hearing concerns, technological knowledge and expectations of online AT. Exit interviews allowed participants the opportunity to provide responses to questions surrounding the online programme, delivery, accessibility and clinician contact. They were also asked questions pertaining to what they enjoyed and did not enjoy about the online AT. The participants were also encouraged to provide any other feedback or comments.

### Data collection

- *Screening*: Potential participants were given consent forms, and after signing the forms they were screened using the Mini-Cog and the Task Ability Questionnaire.
- *Pre-intervention*: Eligible participants were pre-assessed using the APHAB and QuickSIN. The Entrance Questionnaire was also administered. Entrance interviews were conducted and an introductory session including programme demonstration was done to ensure participants knew how to navigate the AT programme.
- *Intervention:* The AT programme was then completed by all participants at their own convenience over 4 weeks, with the researcher monitoring their progress through the LACE Online cloud server. Participants were given a brief overview of their progress weekly through WhatsApp messages (one participant did not have a cell phone; therefore, their results were consented to be sent to their communication partner).
- *Post-intervention:* Upon the completion of the programme each participant was re-assessed using the APHAB and QuickSIN. The SUS and Exit Questionnaires were administered, and the exit interviews were conducted.

The AT programme was completed while the participants were wearing their HAs. The QuickSIN was conducted in an audiometric booth through free field speakers while the participants were wearing their HAs. The QuickSIN was also conducted as part of the AT programme, and the results were captured. All questionnaires and screening tests were conducted under the supervision of the researcher. The complete hybrid tele-rehabilitation programme ran over 6 weeks.

### Data analysis

#### Quantitative data

Descriptive statistics were used for the quantitative data. Descriptive statistics were advantageous as they provided meaningful summaries of the findings, which allowed the intervention modality to be analysed from different aspects (Mishra et al., 2019) (see Table 1 for a brief overview).

**TABLE 1:** Summary of quantitative data analysis.

Data	Analysis method
Number of AT sessions completed (compliance)	Frequency and descriptive tables
Contact time (between the researcher and participant)	
Associated costs of the tele-rehabilitation	
QuickSIN scores	Comparative tables
APHAB scores	
AT (LACE Online) scores	
SUS results	Bar graph

AT, auditory training; APHAB, Abbreviated Profile of Hearing Aid Benefit; QuickSIN, Quick Speech In Noise; LACE, listening and communication enhancement; SUS, System Usability Scale.

#### Qualitative data

Thematic analysis of the qualitative data was conducted to determine any commonality between participant interviews. The thematic analysis in the current study adopted a systematic phased process outlined by Nowell, Norris, White and Moules (2017). The analysis process included:

- *Familiarisation with the data:* Researcher reviewing transcripts.
- *Generating initial codes:* Coding of data and researcher reflexive writing of initial thoughts and impressions of the data.
- *Searching for themes:* Identification of possible themes based on common participant experiences.
- *Reviewing themes:* Determination of link between themes and participant experience.
- *Defining and naming themes:* Definition and clarification of themes and subthemes.
- *Producing report:* Write-up of common themes and subthemes with raw data (participant quotes) embedded within report.

### Ethical considerations

This study followed the ethical principles outlined by the World Medical Association (WMA) in the Declaration of Helsinki to guide researchers in protecting the rights, mental and physical well-being of human participants (World Medical Association 2013). Notable ethical points of the current study included the following:

- Participants were given the study overview in the early stages of recruitment as well as during the first in-person appointment so that they understood what the research entailed, thus allowing them to give informed consent. No one was forced to participate. Participants were also able to withdraw from the study at any time without penalty.
- All participants received the same intervention and were assessed with the same tests.
- Each participant received 1.0 gigabytes (GB) – 1.5 GB of mobile data to allow Internet access.
- Participants received intervention that would have only improved their listening abilities, and there was no risk of further hearing damage.

Ethical approval was obtained from the Human Research and Ethics Committee (HREC) of the University of Cape Town (UCT) (reference number: 642/2018).

## Results

The results are presented according to the objectives of this feasibility study: (1) participant compliance, (2) clinical benefit, (3) participant experience and (4) estimation of costs around hybrid tele-rehabilitation programme implementation.

### Participant compliance

Compliance was determined from (1) the number of sessions completed, (2) contact time between the clinician and participant and (3) the number of participants who withdrew from the programme. The average total completed AT sessions was 9 out of 11 (84.82%). The average total contact time for each participant was 3 h and 25 min, and the weekly average was 22.5 min for each participant. The average number of online interactions was 9, and there were no withdrawals in this study. See Table 2 for the results of (1) and (2).

**TABLE 2:** Compliance factors.

Variable	Pre-intervention	Week 1	Week 2	Week 3	Week 4	Post-intervention	Total (out of 11)	Total
**Number of AT sessions completed per week (1)**								
Participant 1	-	2	3	3	3	-	11	-
Participant 2	-	4	3	2	2	-	11	-
Participant 3	-	0	2	2	2	-	6	-
Mean	-	2	2.67	2.33	2.33	-	9.33	-
**Number of online interactions (telephonic or email contact) (2)**								
Participant 1	1	2	1	2	1	2	-	9
Participant 2	1	2	1	1	1	2	-	8
Participant 3	1	2	2	2	1	2	-	10
**Mean**	**1**	**2**	**1.33**	**1.67**	**1**	**2**	**-**	**9**
**Contact time (face-to-face and telephonic) (in hours) (2)**								
Participant 1	0.75	0.50	0.25	0.25	0.25	0.75	-	2.75
Participant 2	1.50	0.50	0.25	0.25	0.25	1.00	-	3.75
Participant 3	0.75	0.50	0.75	0.25	0.50	1.00	-	3.75
**Mean**	**1.00**	**0.50**	**0.42**	**0.25**	**0.33**	**0.92**	**-**	**3.42**

AT, auditory training.

### Participant benefit (clinical and perceived)

#### Clinical benefit – Online auditory training results and speech in noise perception scores

The results of the AT and QuickSIN showed evidence of clinical benefit with an overall improvement in participants’ listening skills. Table 3 describes each participant’s AT scores for each subtest included in the programme, from the first session compared to the last session. Table 3 also describes the QuickSIN results obtained from participants through the online AT programme and in the audiometric booth (pre- and post-intervention). Table 4 describes the overall mean (AT and QuickSIN scores).

**TABLE 3:** Assessment and intervention results.

Pre-intervention	SNR	Intervention	FirstSession	FinalSession	Improvement (Y/N)	Post-intervention	SNR	Improvement (Y/N)
**Participant 1**								
QuickSIN LACE	15.0 dB	Competing speaker	3.8 dB	6.6 dB	N	QuickSIN LACE	6.5 dB	Y
		Rapid speech	1.2 x	1.5 x	Y			
QuickSIN booth	Left = 1.5 dB	Speech-in-noise training	16.8 dB	8.1 dB	Y	QuickSIN booth	Left = 6.5 dB	N
	Right = -2.5 dB	Word memory training	2.5	4.3	Y		Right = 1.5 dB	N
**Participant 2**								
QuickSIN LACE	0.5 dB	Competing speaker	0.0 dB	−11.7 dB	Y	QuickSIN LACE	9.0 dB	N
		Rapid speech	1.6 x	2.1 x	Y			
		Speech-in-noise training	9.3 dB	−0.3 dB	Y	QuickSIN booth	Left = 8.5 dB	N
QuickSIN booth	Left = 8.5 dB Right = 15.5 dB	Word memory training	1.8	2.1	Y		Right = 13.5 dB	Y
**Participant 3**								
QuickSIN LACE	14.0 dB	Competing speaker	−0.8 dB	−9.9 dB	Y	QuickSIN LACE	−2.0 dB	Y
		Rapid speech	1.2 x	1.3 x	Y			
QuickSIN booth	Left = 10.5 dB	Speech-in-noise training	16.0 dB	−1.5 dB	Y	QuickSIN booth	Left = ---	---
	Right = 12.5 dB	Word memory training	2.2	3.8	Y		Right = ---	**---**

SNR, signal-to-noise ratio; QuickSIN, Quick Speech In Noise; LACE, listening and communication enhancement; dB, decibel.

---, Participant could not be tested – HA battery was not replaced before arriving at assessment.

**TABLE 4:** Assessment and intervention results (mean).

Pre-intervention	SNR	Intervention	First Session	Final Session	Improvement (Y/N)	Post-intervention	SNR	Improvement (Y/N)
**Overall average and mean**								
QuickSIN LACE	9.8 dB	Competing speaker	1.0 dB	−5.0 dB	Y	QuickSIN LACE	4.5 dB	Y
		Rapid speech	1.3 x	1.6 x	Y			
QuickSIN booth	Left = 5.0 dB Right = 6.5 dB	Speech-in-noise training	14.0 dB	2.1 dB	Y	QuickSIN booth	Left = 7.5 dB Right = 7.5 dB	N N
		Word memory training	2.2	3.4	Y			

SNR, signal-to-noise ratio; QuickSIN, Quick Speech In Noise; LACE, listening and communication enhancement; dB, decibel.

#### Perceived benefit – Abbreviated Profile of Hearing Aid Benefit

In this feasibility study, the results of the APHAB showed that with all the participants, there was an overall improvement in perceived HA benefit in three of the four subscales. The average benefit of the RV items improved by 21.3%, BN items improved by 21.2% and AV items improved by 16.4%. The EC average benefit decreased by 1.1%. Table 5 depicts the results of the APHAB.

**TABLE 5:** Abbreviated Profile of Hearing Aid Benefit results.

Pre-intervention	Results	Post-intervention	Results	Improvement (Y/N)
**Participant 1**				
APHAB EC	26.9%	APHAB EC	49.5%	Y
APHAB RV	36.7%	APHAB RV	71.8%	Y
APHAB BN	18.3%	APHAB BN	61.7%	Y
APHAB AV	−82.7%	APHAB AV	−90.7%	N
**Participant 2**				
APHAB EC	11.5%	APHAB EC	12.4%	Y
APHAB RV	2.1%	APHAB RV	33.0%	Y
APHAB BN	4.2%	APHAB BN	−8.3%	N
APHAB AV	−36.7%	APHAB AV	16.5%	**Y**
**Participant 3**				
APHAB EC	82.0%	APHAB EC	55.2%	N
APHAB RV	65.8%	APHAB RV	63.7%	N
APHAB BN	34.7%	APHAB BN	67.5%	Y
APHAB AV	−36.5%	APHAB AV	−32.5%	Y
**Overall average**				
APHAB EC	40.1%	APHAB EC	39.0%	N
APHAB RV	34.9%	APHAB RV	56.2%	Y
APHAB BN	19.1%	APHAB BN	40.3%	Y
APHAB AV	−52.0%	APHAB AV	−35.6%	Y

APHAB, Abbreviated Profile of Hearing Aid Benefit; EC, ease of communication; RV, reverberation; BN, background noise; AV, aversiveness.

### Participant experience of hybrid tele-rehabilitation

#### Entrance Questionnaire

Responses from the Entrance Questionnaire showed that two participants had not had any form of AT before and had also never heard of the concept. Once the concept of the AT included in this study was explained, all participants indicated that they believed they would benefit from it and would also be able to make time for it during their own weekly schedule. All participants indicated WhatsApp to be their preferred method of contact over e-mail, short message service (SMS) and phone call.

#### Exit Questionnaire

According to the results obtained from the Exit Questionnaire, the device most used for the AT was a laptop. The main reason given by participants preferring the laptop was that it was easier to read the text of the programme on the laptop screen compared to the smartphone screen.

#### Exit interviews

The responses from the exit interviews were analysed and formed three different themes and several categories, as depicted in Table 6.

**TABLE 6:** Thematic analysis.

Theme	Category	Description	Example of participant(s) quote
Clinician contact: Helpful and needed	Helpful weekly reminders	All participants found the training enjoyable; however, they did report that they needed the weekly reminders to stay on track to complete the programme. Participant 3 was assisted by her husband with weekly reminders, and he mentioned that it was good to be continually updated; however, he felt pressure to remind her to do her sessions. He concluded by stating that the reminders were a burden, however, very much needed because she struggled to be compliant.	‘The reminders helped me to remember to make time to fit it [*online AT*] in my busy life.’ (Participant 1) ‘Yes, the reminders were helpful … With that [*weekly reminders*], the burden fell on me.’ (Participant 3 – communication partner)
Programme structure	Tasks	There were mixed opinions on the structure of the programme (i.e. task instruction delivery and task order). One participant reported that it was at times difficult to switch from one task to another without warning (e.g. one session may have included a few *Competing Speaker* tasks and a few *Words Memory* tasks). She went on to explain that once she built confidence in one task, the programme moved onto the next task, and it became frustrating.	‘I would get used to doing one task, then the voice would change, and I would have to do something different … I was building confidence in one thing and then it would change … the woman’s voice was the easiest.’ (Participant 1)
	Good session length	In relation to the individual training session length, participants reported no issues with the length.	‘The sessions were not too long…’ (Participant 2) ‘[…*I*]t [*the length*] was fine.’ (Participant 1)
	Helpful communication tips	All participants mentioned, during their interviews, that the communication tips given in the LACE Online were helpful and appreciated.	‘[…*A*]nd I have learnt so much from the tips given in the programme.’ (Participant 1)
Opinions towards AT	Patient education	All participants believed that AT should be included in audiological rehabilitation of hearing loss. Participants noted that patients should at least be informed about the concept of AT so that they know it exists and that it can help with listening skills.	‘They [*public health patients*] should at least be informed about it [*AT*].’ (Participant 1) ‘Yes [*inclusion of AT in regular audiological management*], especially when they can’t hear in noise.’ (Participant 3)
	Enjoyable training	It was apparent from the positive responses given by the participants that they preferred to do the AT sessions in their own time, as there was no pressure to attend regular hospital appointments.	‘I highly recommend this programme … It is a fantastic programme.’ (Participant 1)

LACE, listening and communication enhancement; AT, auditory training.

#### Usability of hybrid tele-rehabilitation programme

**FIGURE 2:**
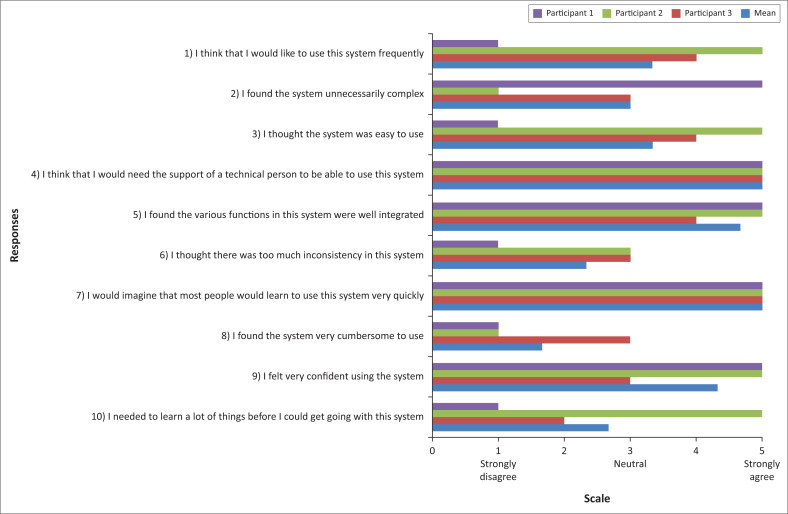
System usability scale responses.

A notable finding from the SUS was that all participants indicated that they believed people would need initial technical assistance to be able to learn to use the programme quickly. Figure 2 depicts the responses.

### Hybrid tele-rehabilitation programme associated costs

The estimated cost of the implementation of the hybrid tele-rehabilitation programme was calculated by including the following costs for each participant:

- LACE Online access – $79.00 (R1200.80)
- mobile data – R80.00 – R100.00
- travel – R28.00 – R30.00.

Table 7 describes the breakdown in costs of the hybrid tele-rehabilitation programme implementation.

**TABLE 7:** Breakdown of costs.

Participants	Breakdown of costs			
	LACE Online (in rands)	Mobile data (in rands)	Travel (in rands)	Total (in rands)
Participant 1	1200.80	80.00	28.00	1308.80
Participant 2	1200.80	80.00	30.00	1310.80
Participant 3	1200.80	100.00	30.00	1330.80
Participant 4	1200.80	†	†	1200.80
Participant 5	1200.80	†	†	1200.80

**Total**	**6004.00**	**260.00**	**88.00**	**6352.00**

LACE, listening and communication enhancement.

† , Participants 4 and 5 did not arrive for their appointments and therefore could not be included in the intervention; however, their costs of online access to the AT programme were still incurred.

## Discussion

This study was, to the knowledge of the researchers, the first to investigate the feasibility of hybrid tele-rehabilitation implementation in South African public hearing healthcare using a hybrid tele-audiology model. Even though the sample size was limited, the feasibility intent of the study sought to investigate the initial findings from a hybrid tele-rehabilitation programme. Thus, the findings from this feasibility study may be seen as initial positive indicators that audiological rehabilitation of disabling hearing loss could be administered using a hybrid model in a low-resourced context. Key findings of this feasibility study indicated a high compliance rate (84.82%) to hybrid tele-rehabilitation, minimal clinician contact time at 3 h 25 min over a 5–6-week period, clinical benefit and positive feedback from participants and programme costs at an estimated R1350.00 per person. Moving forward in the context of COVID-19, using hybrid tele-rehabilitation may be a reality for most audiologists where the relevant resources (e.g. device access) are available.

### Hybrid tele-rehabilitation compliance and clinician contact

The compliance rate for the hybrid tele-rehabilitation programme for this feasibility study was high (84.82%), and this is comparable to the compliance rates noted by Chisolm et al. (2013) (84%) and Tye-Murray et al. (2012) (> 90%). The high compliance rate in this study may be attributed to the hybrid tele-audiology model used where there was regular clinician support. This is an important finding to consider as it implies that tele-rehabilitation should not fully replace clinician contact; in fact, clinician contact (through a hybrid model of delivery) is pertinent to positive patient compliance. A similar finding of clinician contact positively impacting patient compliance was also reported by Chisolm et al. (2013) and Tye-Murray et al. (2012).

The total clinician time in this feasibility study was at an average 3 h and 25 min over 6 weeks for a full AT programme. In a usual audiology clinic, 3 h would equate to 3–4 diagnostic tests or initial HA fittings. This indicates that a hybrid model of tele-rehabilitation delivery may be introduced into the existing audiology clinics in low-resource contexts without adding a significant workload burden for the clinicians. Although the AT programme was explained in detail, and participants were given a document summarising how to log in; patients required further reassurance and support in week 1. This finding is similar to a crucial point made by Nemes (2010), who suggested that tele-audiology methods should be applied, without disregarding duties towards patient support in the form of clinician contact. In addition, Sweetow and Henderson-Sabes (2010) highlighted that compliance with home-based AT has been reported by many audiologists to have improved with an initial face-to-face introductory session with the clinician. Considering these previous findings and the findings of the current study, it can be said that hybrid tele-rehabilitation has shown to improve (1) compliance, (2) patient trust and (3) patient empowerment (Nemes, 2010; Sweetow & Henderson-Sabes, 2010; Tye-Murray et al., 2012).

A hybrid model may also address issues with low follow-up rate, as noted by previous studies (Chisolm et al., 2013; Ramkumar, Hall, Nagarajan, Shankarnarayan, & Kumaravelu, 2013; Scott & Mars, 2015). The findings of this feasibility study suggest that adult HA patients may comply with online AT as it is convenient and does not require regular travel to the hospital, which implies, for most low-resourced countries, taking a day off work. Furthermore, the high compliance rate of this feasibility study is in line with recent research in South Africa (Ratanjee-Vanmali et al., 2020).

### Clinical benefit and participant experience

#### Clinical benefit

The communication skills of HA users are multifaceted and need comprehensive assessment which assists in measuring improvements and establishing communication goals (Sweetow & Henderson-Sabes, 2010). Measuring self-perceived benefit should be considered important when administering AT – as seen with the results of the APHAB in the current study, which aligned with the AT training effects. The results of the APHAB showed that the BN subscale was one of the most improved subscales overall. This is an important result, as one of the key training tasks of the LACE programme is speech-in-noise training. Even with the results of the EC subscale of the APHAB failing to improve post-intervention shows that it could be attributed to the AT. This is because AT focuses on improving listening in noise skills and the EC items all address quiet situations, and therefore the perceived benefit could have been more perceptible in the other subscales which deal with complex noisy situations. An improvement in listening in noise skills would then determine how the participants scored their listening skills in noisy environments (e.g. BN subscale) compared to quiet environments (EC subscale). This finding suggests that the results of an AT programme should be cross-checked with a self-perceived benefit scale, such as the APHAB.

#### Participant experience

Participants responded positively to the hybrid tele-rehabilitation according to the interviews, which was a promising indicator that South African public health patients may be open and willing to engage in tele-rehabilitation. All participants indicated that AT should be included in audiological rehabilitation. Some stated that patients should, at least, be informed about it, citing that they had never heard of AT before this research. The lack of information and implementation of further hearing rehabilitation could be because public health audiologists are challenged with attending to back-to-back patients and the diagnosing and fitting of patients leaves little time for further services (e.g. AT) (Makhoba & Joseph, 2016; Pienaar et al., 2010). Makhoba and Joseph (2016) noted that the inclusion of communication strategies was the only form of further audiological rehabilitation after HA fitting, and it was only reported to be included by 69.8% of their sample. The lack of AT inclusion is further corroborated by other international studies which have reported that AT is a vital yet underutilised tool in the field of audiology (Chisolm et al., 2013; Dubno, 2013; Ferguson & Henshaw, 2015; Sweetow & Palmer, 2005). Therefore, the use of online AT whereby the patients receive a self-explanatory document (e.g. instruction pamphlet) may afford public health patients the opportunity to receive AT, without placing a burden on public health audiologists. As found in the current study, it would not require a substantial amount of time to explain AT and introduce patients to an online programme (approximately 15 min) during their first obligatory HA follow-up.

### Hybrid tele-rehabilitation costs

A factor in assessing the feasibility of the hybrid tele-rehabilitation programme is the determination of associated costs. These costs generally include the costs of resources (e.g. assessments, programme access, etc.), health facility costs (e.g. equipment and health professionals) and patient costs (e.g. travel expenses and Internet data costs). The current study reviewed the costs of the hybrid tele-rehabilitation programme from a health provider perspective and included the costs of the American product (LACE Online), which was influenced by the dollar to rand exchange rate, mobile Internet data and patient travel expenses. The total costs for this feasibility study were R6352.00, and the average cost per participant was R1350.00.

#### Internet-capable device access in low-resource contexts

Another important factor to consider with tele-rehabilitation is patient access to Internet-capable devices. It is worth noting that Internet connectivity and device access in previous literature was anticipated to be a challenge in implementation and compliance to tele-audiology methods in low-resourced contexts (Clarke & Mars, 2015; Monica et al., 2017; Ramkumar et al., 2013). However, this feasibility study highlighted that even though most patients who seek public healthcare may be presumed to be from low socio-economic backgrounds, their access to technology and an Internet-capable device was not observed to be limited. Internet connectivity and device access is increasing in low-resource contexts, and Rutherford and Petersen (2019) noted that the penetration of the smartphone market in South Africa is 73%, which is arguably significant and holds promise for tele-audiology applications.

### The COVID-19 safety benefits of hybrid tele-rehabilitation

Physical distancing and mandatory face coverings decrease the risk of infection but increase breakdowns in communication, which is comparatively more severe in those living with hearing loss because they are unable to read lips or facial cues (Naylor, Burke, & Holman, 2020). To counter these affects, some audiologists adopted transparent masks or face shields where in-person appointments are a necessity. However, current national laws and social responsibility noted that where services could be provided using socially distant tele-audiology methods, audiologists were encouraged to do so. The dilemma which many audiologists faced was the uncertainty around tele-audiology methods; this included which services to provide using online methods, efficiency of services and, most importantly, the question of patient benefit (Nemes, 2010). A recent international survey showed that audiologists’ attitude towards telehealth during COVID-19 was overall positive, with some hesitancy around certain factors – mainly, time and financial constraints, patient technology access and technology knowledge (Eikelboom et al., 2021). The previously outlined patient and clinician benefits from the current study, which included time and cost savings, are some of the possible broader advantageous factors of implementing hybrid tele-rehabilitation. When considering the current COVID-19 pandemic and its impact on everyday practices, the benefits may be expanded to include that it is a COVID-19 safe option which minimises close contact and thus reduces the risk of infection.

### Study strengths

#### Original research

The current study was, to the knowledge of the researcher, the first to investigate the feasibility of implementing tele-rehabilitation through an online AT programme in a South African public health context. With regard to tele-audiology research in South Africa (and globally), most studies aim to include screening or diagnostic testing in the scope of hearing healthcare service provision. This feasibility study on tele-rehabilitation presented research on a topic that is not widely researched in the field of audiology and would therefore add value to the larger pool of audiology research.

#### Relevant research

With the growing influence of telehealth applications in the field of audiology, the current feasibility study presents a relevant topic. South Africa is seen as one of the leading countries in tele-audiology research and application because of the significant need for cost-effective, efficient solutions for its current public health system. In terms of audiological rehabilitation service provision in South Africa, research efforts need to be directed at looking into methods that can provide more comprehensive services to public health patients in a manner that would not additionally burden the current patient load.

#### Mixed methods

The current study included both quantitative and qualitative measures. It is important to include patient experience when considering the feasibility of a new service delivery model for patients as the quantitative factors can be limited in its means to show patient satisfaction and self-perceived benefit. Also, mixed methods allowed for comprehensive data collection from different aspects of the intervention modalities.

### Study limitations

#### Sample size

The main limitation of this feasibility study was the sample size. The sample size in the current study was mostly influenced by the design of previous research, recruitment challenges and the objectives of this feasibility study. Regarding previous research with AT and given the nature of AT, small sample sizes have been preferred (Dubno, 2013; Ferguson & Henshaw, 2015; Santos et al., 2014; Sweetow & Henderson-Sabes, 2004). A lack of control group as well as the nonrandomisation of the sample was a bias threat. However, the feasibility approach of the current study was not suitable for participant randomisation.

The intention of including inferential statistics alongside the descriptive statistics could not be accomplished because of the sample size. Statistical inferences would not have been valid with the current sample size. The exclusion of inferential statistics implied a limitation on the interpretation of the results and no concrete conclusions could be made to generalise or make inferences of the study findings; however, the results of the current study may be used as a foundation for comparison to future large-scale research.

## Conclusion

The results of the current study indicated that the use of a hybrid tele-audiology model to provide audiological rehabilitation (such as online AT) may be feasible for implementation in low-resourced contexts like South Africa, with minimal additional costs, workload and time (face-to-face clinical contact) implied in current services. However, limitations of this study included the sample size which restricted generalisability. Therefore, the findings of this study can be seen as positive indicators for the use of hybrid telehealth as a modality for the delivery of audiological rehabilitation in low-resourced contexts during and after the COVID-19 pandemic, but further large-scale research is still needed.
